# Nanoporous Anodic Alumina Platforms: Engineered Surface Chemistry and Structure for Optical Sensing Applications

**DOI:** 10.3390/s140711878

**Published:** 2014-07-07

**Authors:** Tushar Kumeria, Abel Santos, Dusan Losic

**Affiliations:** School of Chemical Engineering, Engineering North Building, The University of Adelaide, North Terrace Campus, Adelaide SA 5005, Australia; E-Mails: tushar.kumeria@adelaide.edu.au (T.K.); dusan.losic@adelaide.edu.au (D.L.)

**Keywords:** nanoporous anodic alumina, surface modification, optical biosensor, plasmon resonance, reflective interferometry

## Abstract

Electrochemical anodization of pure aluminum enables the growth of highly ordered nanoporous anodic alumina (NAA) structures. This has made NAA one of the most popular nanomaterials with applications including molecular separation, catalysis, photonics, optoelectronics, sensing, drug delivery, and template synthesis. Over the past decades, the ability to engineer the structure and surface chemistry of NAA and its optical properties has led to the establishment of distinctive photonic structures that can be explored for developing low-cost, portable, rapid-response and highly sensitive sensing devices in combination with surface plasmon resonance (SPR) and reflective interference spectroscopy (RIfS) techniques. This review article highlights the recent advances on fabrication, surface modification and structural engineering of NAA and its application and performance as a platform for SPR- and RIfS-based sensing and biosensing devices.

## 1. Introduction

In recent years, an increasing amount of research has been focused on exploring the production of novel nanoporous materials and their application for developing highly sensitive chemical and biosensing devices. These novel analytical devices based on nanoporous materials are cost-effective, highly sensitive due to the large surface-to-volume ratio, and additionally show excellent selectivity when coupled to biorecognition elements [1]. In particular, nanoporous materials synthesized by electrochemical techniques such as porous silicon (pSi), nanoporous anodic alumina (NAA), and titania nanotube arrays (TiNTs) are the top choice of researchers as chemical and biosensing substrates [2,3]. These nanoporous materials have been combined with various optical (e.g., surface plasmon resonance, reflective interference, optical waveguiding, Raman spectroscopy and others) and physical (e.g., electrochemical or impedance technique, piezoelectric, surface acoustic wave and so on) detection methods to develop highly innovative and capable analytical tools [3]. Amid these nanoporous materials, NAA prepared by anodization of aluminum foil, possesses outstanding chemical, optical, and mechanical properties such as chemical resistance, thermal stability, hardness, biocompatibility and large specific surface area. Particularly, its high specific surface area to volume ratio is dearly useful to enhance optical signals when target molecules (analytes) are attached inside the nanopores [3,4]. Therefore, NAA is an excellent platform to develop sophisticated and relevant applications as selective molecular separation, chemical/biological sensing, catalysis, cell adhesion and culture, data storage, energy generation and storage, drug delivery and template synthesis [3–6]. These properties make NAA substrates excellent platform for developing advanced, smart, simple and cost-effective analytical devices. In addition, the ability to engineer its pore size, geometry, and surface chemistry make NAA even a more versatile nanomaterial as these features can be tuned in order to integrate separation and sensing capabilities into all-in-one devices [3]. Recently, the use of NAA for chemical and biosensing applications has gained a very fast pace with potential for future commercialization. The perfectly organized nanoporous structure of NAA, its optical activity and the ability to modify its structure and chemistry have resulted in its vast application as a substrate for surface plasmon resonance and reflective interference-based detection systems [3]. However, no literature review has highlighted and summarized these developments.

Herein, for the first time, we review the major advances and developments of chemical and biosensing systems based on NAA in combination with surface plasmon resonance (SPR) and reflectometric interference spectroscopy (RIfS). First, we present the fundamental aspects of the fabrication and structure of NAA prepared by electrochemical anodization of aluminum. Next, recent advances on the modification of the surface chemistry of NAA using various techniques are reported. Then, we present the most outstanding works in structural engineering of NAA relating aimed at engineering its optical properties. Finally, we present examples of recent advances on SPR and RIfS chemical and biosensing systems based on NAA, providing details of their principles, performance and practical applications.

## 2. Fabrication and Structure of NAA

**Figure 1.**
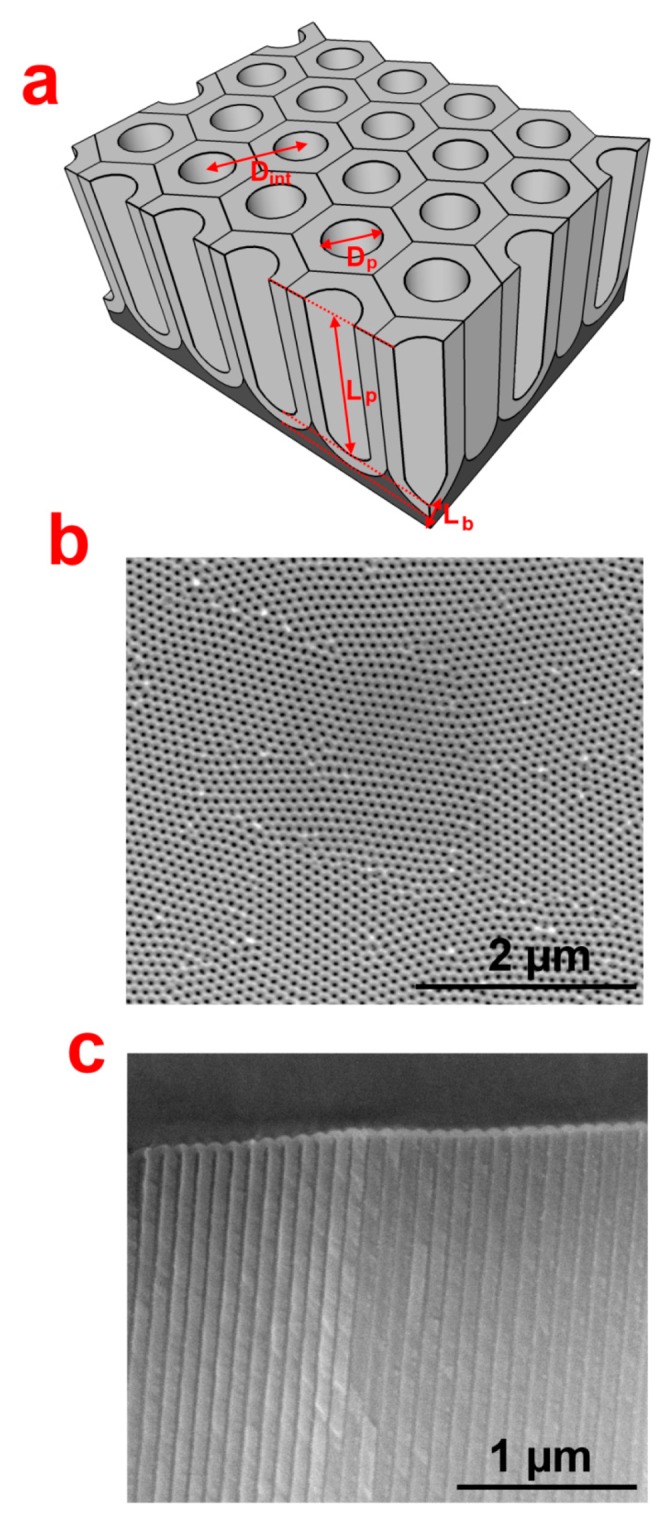
(**a**) Schematic describing the structural characteristics of NAA (*i.e.*, pore diameter: D_p_, pore length: L_p_, barrier layer thickness: L_b_, and interpore distance: D_int_); (**b**) Top and (**c**) cross-sectional SEM images of NAA.

Aluminium oxide has been used since the early 1900s due to its chemical and corrosion resistance properties. Keller *et al.* in 1953, for the first time, described the structure of anodically grown alumina as a close-packed hexagonally organized duplex structure with a porous and a barrier type oxide layer [7]. In general, the structure of self-organized NAA can be described as a matrix of alumina with close-packed arrays of hexagonally-arranged cells with a cylindrical pore at its center that grows perpendicularly to the surface of the aluminum substrate [8]. It is noteworthy that during anodization an electrochemical equilibrium between the rate of oxide growth and its dissolution through the barrier layer at pore bottom is achieved, which is essential for steady state growth and hexagonal packing of nanopores [9,10]. Free-standing NAA substrate can be obtained by selectively etching the underlying aluminum after anodization. Membrane type NAA can be produced by further etching the oxide barrier layer at the pore bottom. This results in membranes with vertically-straight nanochannels. The key structural parameters of NAA are pore diameter (D_p_), inter-pore distance (D_int_), pore length (L_p_) and oxide barrier layer thickness (L_b_). Figure 1 presents a schematic of a typical NAA structure along with scanning electron microscopy (SEM) images of its top and cross-sectional surface. The structural features of NAA (*i.e.*, D_p_, D_int_, L_p_, and others) can be precisely controlled by the anodization conditions. In this regard, the geometric features of NAA can be varied in the range of 10–400 nm for pore diameter, 50–600 nm for inter-pore distance, from several nanometers to hundreds of micrometers for pore length, and 30–250 nm for the oxide barrier layer thickness. Other important characteristic parameters of NAA are its pore density (δ_p_) and porosity (P), which can be obtained in a range between 109–1011/cm^2^ and 5%–50%, respectively [9,11]. During the last decades, the effect of the anodization conditions on the structural features of NAA and self-organizing of pores has been extensively investigated. Anodization parameters such as the anodization voltage, electrolyte type, concentration and temperature have been recognized to be the most critical to control the self-ordering process and the geometry of the resulting NAA structures [11–13]. Aqueous solutions of sulphuric acid (H_2_SO_4_), oxalic acid (H_2_C_2_O_4_) and phosphoric acid (H_3_PO_4_) are the most commonly used electrolytes for preparation of NAA by conventional anodization process at voltages of 25, 40, and 195 V, respectively [14–16]. This process is called “mild” anodization (MA) as moderate voltages and temperatures are used during the anodization. Other acids have also been reported to yield self-organization of NAA pores, which include aqueous solutions of citric, malic, malonic, tartaric and sulfamic acids [12,17–21]. However, the pore organization achieved with these acid electrolytes is poor in comparison to the conventionally used acids (*i.e.*, sulphuric acid, oxalic acid, and phosphoric acid). Each acid electrolyte has its respective optimum anodization voltage and temperature, which leads the pore growth under self-organizing conditions [22]. Self-organizing conditions for different electrolytes are provided in Table 1

The major milestone in the history of NAA fabrication was reported in 1995, when Masuda and Fukuda successfully prepared highly self-organized NAA with very narrow size distribution and extremely high aspect ratios by using a two-step anodization process [14–16]. In this approach, the porous oxide layer obtained by first anodization is selectively removed to pre-structure the aluminum (Al) surface. This leads to perfect self-organization of pores from top to bottom during the second anodization step. Pre-structuring of Al surface improves the pore arrangement during two/multi-step anodization. However, the slow pore-growth rate in the range of 2–7 μm/h (depending on the electrolytic conditions) is an inherent disadvantage of mild anodization. This problem was solved by a new anodization approach called “hard” anodization (HA) introduced by Gösele and co-workers [8]. Under HA conditions (*i.e.*, high voltages and low temperatures) considerably higher pore-growth rates in the range of 50–100 μm/h could be attained. These two anodization regimes (*i.e.*, MA and HA), under different electrolytic conditions combined with chemical etching, provided new approaches to engineer the structure of NAA with desired pore dimensions and shapes. Several strategies like periodic anodization profiles (voltage or current) with or without replacement of the acid electrolyte have been successfully explored to fabricate NAA with complex pore geometries. These electrochemical approaches have made it possible to fabricate NAA with different pore morphologies, including funnel-type, branched pores, periodically-shaped pore structures, and hierarchical and multi-structured pores. These nanostructures have been demonstrated using the aforementioned anodization and post-synthesis treatment processes [24–28]. This ability to structurally engineer the pores of NAA *ad lib* in combination with current surface modification methods provides new tools to modify and improve the properties of NAA for developing highly sensitive and selective chemical and biosensing systems. Notice that highly ordered NAA structures can be produced by other nanofabrication approaches, which have been established to precisely control the growth of NAA. Some of them are stamp imprinting and lithography techniques (*i.e.*, colloid sphere lithography, electron-beam lithography, focused-ion beam lithography, holographic lithography, direct laser writing lithography and Ar plasma etching). The choice of the fabrication technique depends on the scale of required pore arrangement, grade and arrangement of organization and pore size and shape. The structural (*i.e.*, pore diameter, pore length and inter-pore distance) and chemical (*i.e.*, distribution and content of impurities, crystallographic phase) properties of NAA are crucial in determining its optical properties. Thus, the fabrication and structural engineering become the key factors when designing optical sensing devices based on NAA.

## 3. Properties, Surface Chemistry and Surface Functionalization of NAA

### 3.1. Properties of NAA

NAA is a magnificent platform for developing chemical and biosensing devices due to its distinctive physical and chemical properties. Optical characteristics of NAA such as its photoluminescence (PL), transmittance, reflectivity, and absorbance can be used as principle of detection in highly sensitive and selective chemical and biosensing tools [3]. The pores of NAA can also act as container for holding chemical and biological payloads/molecules that take part in recognition reaction. Another advantage of NAA is that all the microfabrication and miniaturization techniques available for silicon technology can be readily employed for fabricating NAA structures. In this regard, the generation of micropatterns on NAA structures is very useful approach for the development of microfluidics and microarray-based sensing devices. It is worth stressing that the presence of impurities in the inner layer of pores (*i.e.*, close to the pore center) and high density of hydroxyl groups make functionalization of NAA very simple using various techniques (e.g., chemical vapour deposition, sol-gel, dip coating, self-assembly, atomic layer deposition and others). These processes can be used to endow NAA with chemical/physical selectivity toward target molecules (e.g., antigens, DNA, proteins, and enzymes) [29]. These modifications can specifically be aimed at enhancing a wide range of properties of NAA such as its reflectivity, hydrophobicity or hydrophilicity, anti-fouling, and chemical resistance, depending upon its intended application.

### 3.2. Surface Chemistry and Surface Functionalization of NAA

**Figure 2.**
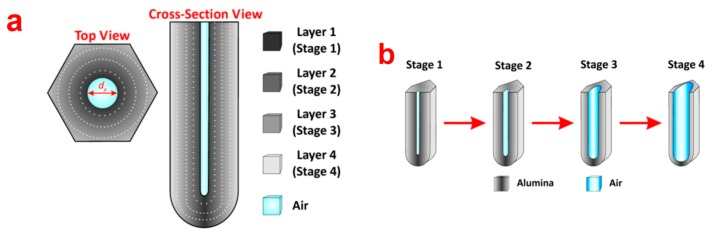
(**a**) Schematic of top and cross-sectional view showing the distribution of impurities in a NAA pore cell; (**b**) Four different stages of dissolution of NAA alumina layer under acidic conditions (5 *v* % H_3_PO_4_ at 35 °C) (adapted with permission from reference [32]).

NAA possess a layered surface chemistry with electrolytic impurities distributed in an onion-like layered manner. It is intended that the chemical structure of NAA is composed of an outer layer close to the center of pore and an inner layer away from the central pore. The outer layer is composed of aluminium oxide (Al_2_O_3_) contaminated with acid electrolyte impurities and the inner layer is mainly composed of pure Al_2_O_3_ [22]. This has been proven previously by several investigations, however, the number of layers reported by each study is different. Thompson *et al.* argue that chemical structure of NAA has two layers of onion-like structure where the outer layer is contaminated with anionic species form electrolyte and the inner layer is dense pure alumina [30]. However, there are other studies that point out that the chemical structure of NAA actually consists of more than two layers. As for this, Yamamoto *et al.* revealed that the chemical structure of NAA is composed of three chemical layers as indicated by its photoluminescence spectrum after specified chemical etching steps [31]. A recent study by our group on the other hand suggests that the real chemical structure of NAA is composed of four onion-like layers with decreasing electrolytic impurities from the outer to the inner layer [32]. A schematic of the chemical structure of NAA is provided in Figure 2.

**Figure 3.**
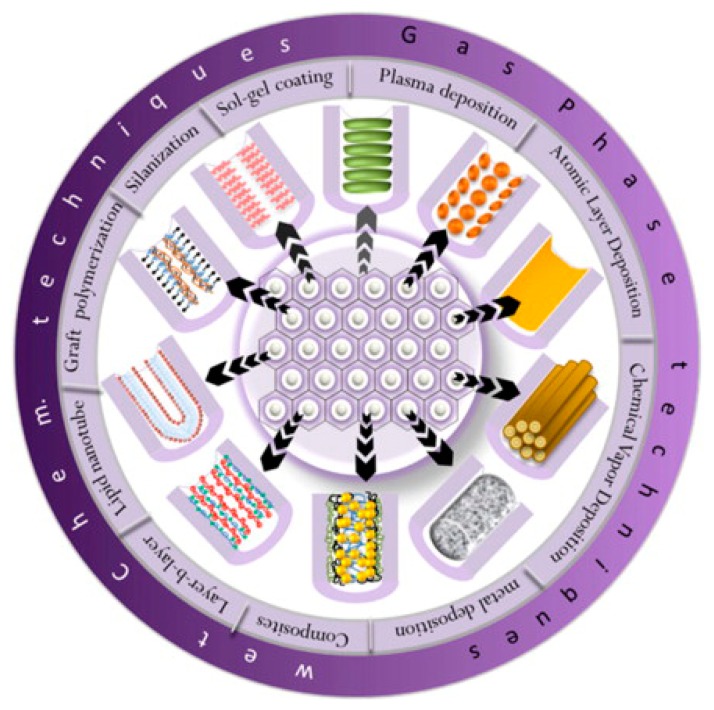
A summary of typical wet chemical and gas phase techniques used to modify the surface of NAA (reprinted with permission from [29]).

The chemical dissolution of NAA was monitored in real-time by reflectometric interference spectroscopy and changes in effective optical thickness (*OT_eff_*) and intensity of the *OT_eff_* peak lead to the aforementioned conclusion. A vast number of surface modification techniques have been developed to protect NAA from acidic environment and impart specific functionalities to its surface. These techniques can be divided into two main categories as physical/gas-phase deposition (*i.e.*, thermal vapor deposition, chemical vapor deposition (CVD), plasma polymerization and atomic layer deposition (ALD)) and chemical modifications (*i.e.*, self-assembly processes of silanes, organic and phosphonic acids, layer-by-layer deposition, polymer grafting, sol–gel processing, electrochemical and electroless deposition) [29]. Subsequent modifications of these surface functionalities of NAA can be used for either to prepare other functional nanomaterials (e.g., nanorods, nanoparticles, nanotubes) or attach biomolecules inside the pores of NAA. The most commonly used approaches for modifying the surface of NAA are described in Figure 3. Further control on the functionalities of NAA surface can be obtained by using a combination of any of these modification techniques on NAA for specific applications.

#### 3.2.1. Gas-Phase Techniques

Gas-phase functionalization techniques including thermal vapor deposition, sputtering, pulsed laser deposition, chemical vapor deposition and plasma polymerization are employed to deposit a wide variety of materials as metals, metal oxides, nitrides, and carbon nanotubes inside NAA [33]. These methods can be used to tune the properties of NAA for specific applications, the most important examples of which are summarized in the following sections.

##### Metal Coating Using Thermal Vapor, Sputtering and Electron-beam Deposition

Metals films including gold, silver, platinum, palladium, titanium and nickel are generally deposited on NAA using thermal vapor deposition technique. These films are aimed at improving conductivity, reflectivity and chemical stability of NAA substrates [33]. Coatings of metals such as Au, Pd, Pt and Ti are reportedly used to improved catalytic properties of NAA, whereas Ni or Co coatings impart magnetic properties to NAA substrates. Metal coatings also serve as the basis for further chemical modification of NAA to help in binding various chemical and biological species with particular relevance for optical sensing and molecular separation applications [29]. The main disadvantage of these techniques is that the penetration depth is limited and only the top surface of NAA is modified.

**Figure 4.**
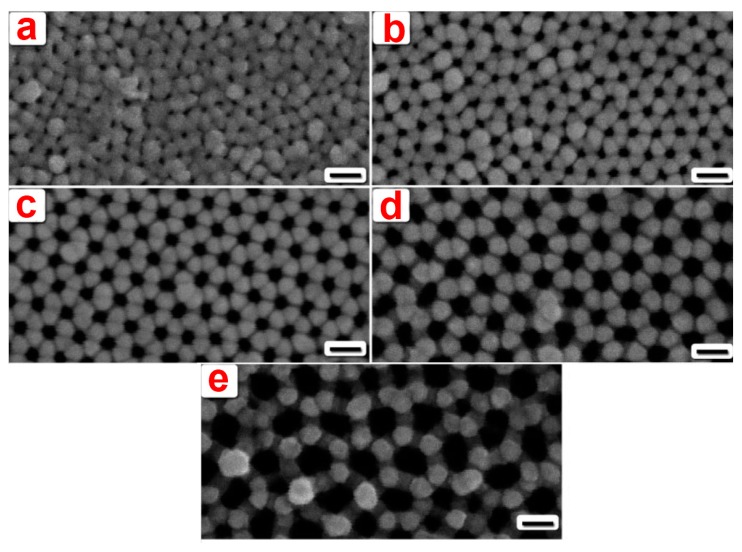
SEM images of DC-magnetron Ag sputter-coated NAA membranes fabricated at different voltages and sputtering time set to 10 min (scale bar = 100 nm): (**a**) 20 V; (**b**) 30 V; (**c**) 40 V; (**d**) 50 V; (**e**) 60 V. (Adapted with permission from [37]).

Gold coatings on NAA substrates have been used as a base layer for layer-by-layer assembly of α,ω-diorganophosphonate/zirconium(IV), which plays a crucial role in confining LBL multilayers inside the pores and preventing them from being suspended over the top of the NAA substrate [34]. Platinum coatings were used by Toh *and coworkers* to apply electric field to the NAA membrane for separation of charged proteins. Additionally, the same group achieved size-selective separation by increasing the thickness of the sputtered Pt layer [35,36]. Hexagonal arrangements of NAA pores has been exploited by several groups to prepare surface enhanced Raman scattering (SERS) substrates by depositing noble metals on it [37,38]. Qui *et al.* deposited an Ag layers on the top surface of NAA templates using a direct-current magnetron sputtering. The resulting metal caps on the surface act as Raman hot spots (Figure 4) [37]. This nanocap SERS-active hot-spot structure provides new opportunities for the fabrication of robust, exceptionally sensitive, cost-effective and large-area chemical and biological sensors. Béron *et al.* fabricated highly ordered patterned Permalloy nanometric structures by depositing Fe-Pd using ion beam sputtering on top of an anodic aluminium oxide nanoporous template. Their micro-magnetic simulations indicate the presence of Permalloy on one side of the pores, forming an anisotropic nanopillar array combined with an antidot array [39,40].

##### Plasma Polymer Deposition

**Figure 5.**
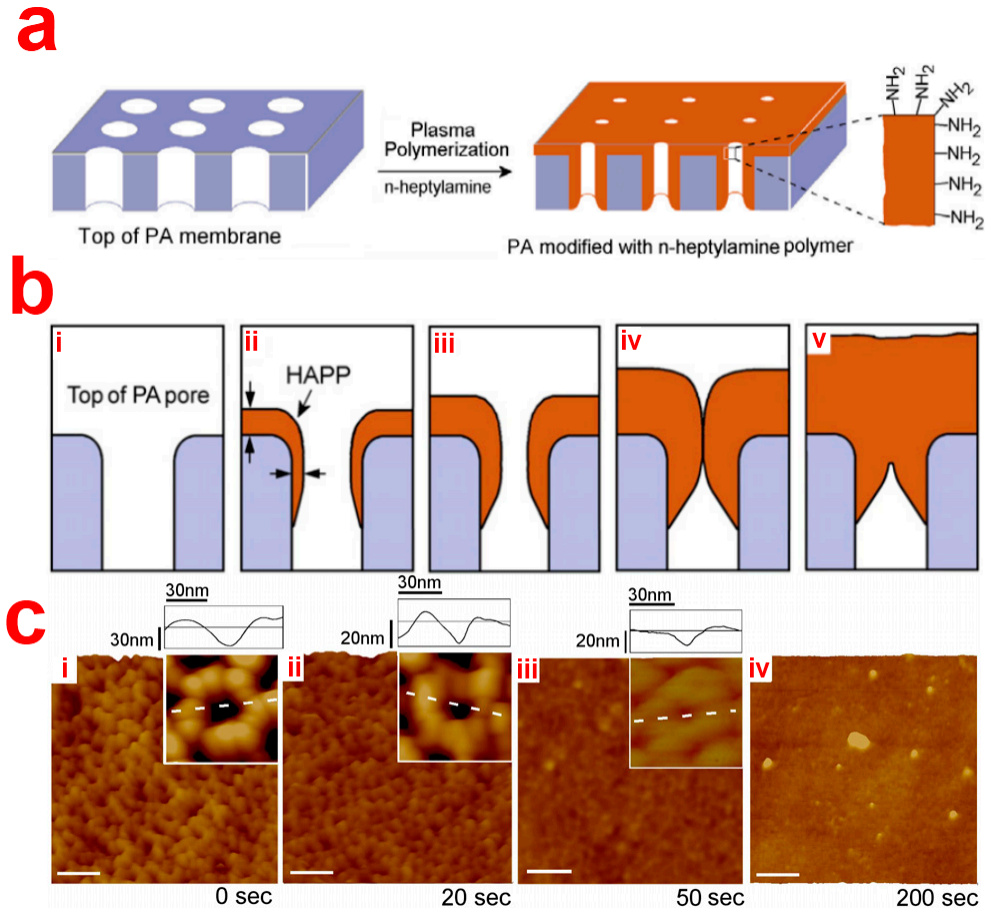
(**a**) A schematic illustration of n-heptylamine coating on NAA; (**b**) Scheme showing the shadowing effect resulting in blocking of pores with long time deposition; (**c**) AFM images of plasma-coated NAA for different time periods. (Adapted with permission from [42]).

Plasma polymerization can be used to deposit reactive and biocompatible polymer films with controlled thickness and chemical functionality with a wide range of functional groups, including amine, carboxyl, hydroxyl, epoxy and aldehyde groups. Brenov *et al.* for the first time, reported on the modification of NAA by plasma polymerization [41]. They prepared Janus-type NAA membranes by depositing hydrophobic fluorocarbon polymer from plasma polymerization of C_4_F_8_ on one side of the NAA membranes while the other side was left unmodified. Water contact angle (WCA) on the hydrophobic side of the NAA membranes was reported (WCA) 150°, while the other side had a WCA lower than 20°. Additionally, the pore diameter on the functionalized side of the NAA membranes was reduced from 160 to 80 nm. In order to improve both structural and surface properties of NAA membranes, Losic *et al.* functionalized their top surface by plasma polymerization of *n*-heptylamine. They were able to precisely tune the pore diameter of NAA from 20 nm to <5 nm by adjusting the time duration of the plasma deposition [42]. The process yielded an amine terminal rich surface. However, it is worthwhile noting that this functionalization approach is limited to surface-based applications due to the poor depth penetration inside the nanopores of NAA (Figure 5).

##### Atomic Layer Deposition (ALD)

**Figure 6.**
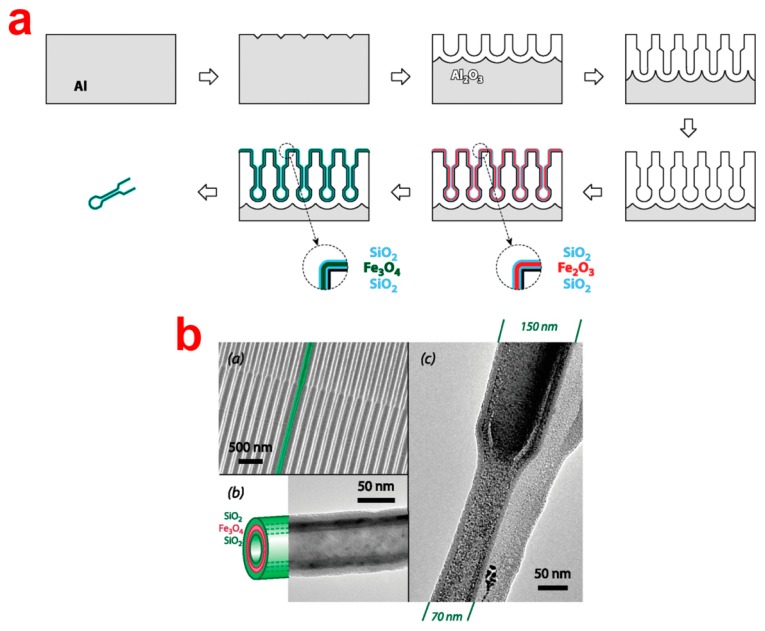
(**a**) A schematic illustration of fabrication of modulated pore NAA and subsequent deposition of magnetic nanotubes; (**b**) SEM and TEM images of the prepared magnetic nanotubes. (Adapted with permission from [53]).

ALD enables the precise deposition of a broad range of materials, including oxides, nitrides, sulfides and metals coatings. The resulting coatings present high mechanical, chemical and thermal stability, and optical activity. This technique has been extensively exploited for modifying the inner surface of NAA as a result of its penetration distance inside nanopores. Studies on ALD modification of NAA demonstrate that silica, titania and alumina can be controllably deposited inside NAA pores to reduce its pore dimensions, improving its catalytic, optical and transport properties and generate 1D nanostructures [43–46]. In this regard, Velleman *et al.* deposited silica (SiO_2_) inside NAA by ALD method to reduce the pore diameter and subsequent modification with specific silane chemistry, which endowed NAA with chemical selectivity towards transporting dye molecules [5]. In addition, ALD has been used to prepare ultra-high aspect ratio nanotubes/wires of various materials, including pure metals (Cu, Ni, and Co) and their oxides (e.g., ZnO, TiO_2_, and ZrO_2_) [46–49]. For example, highly uniform, densely packed and vertically aligned TiO_2_ nanotubes were fabricated by ALD inside NAA templates using TiCl_4_ as a precursor. The NAA template was then chemically removed to yield nanotubes with perfectly controllable tube diameter, spacing and wall thickness [50]. In a similar manner, ZnO-nanowires were produced by ALD deposition inside NAA templates [51]. Bachmann and co-workers fabricated magnetic nanotube arrays in NAA templates with modulated pores by means of depositing Fe_3_O_4_ via ALD using ferrocene and ozone as precursors (Figure 6) [52,53].

##### Chemical Vapor Deposition (CVD)

**Figure 7.**
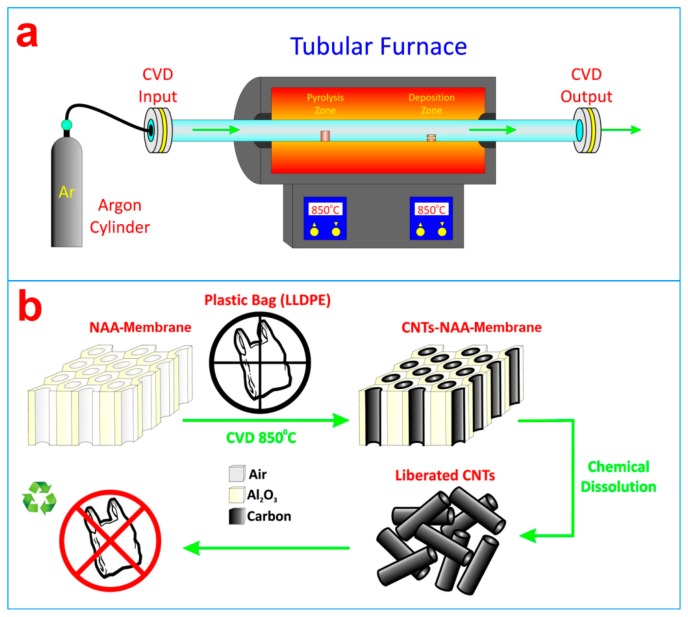
(**a**) A scheme showing the setup used to grow CNTs inside NAA templates by CVD; (**b**) Schematic illustration of recycling process of plastic by CVD process to yield CNTs. (Adapted with permission from [58]).

Chemical vapor deposition (CVD) involves dissociation of gaseous molecules by heat, light or plasma to form stable and conformal films on a substrate. CVD is mostly used to grow carbon nanotubes (CNTs) inside NAA pores [54–56]. This process provides vertically aligned arrays of CNTs with controlled dimensions. The carbon layer grows inside the NAA pores via pyrolysis of carbon source (mainly acetylene gas) at elevated high temperature (>600 °C) for varying time [57]. A wide range of carbon sources (either as liquid, gas or solid) have been used for growing CNTs. Recently, our group demonstrated the ability of this technique to recycle plastic waste and convert it into CNTs inside NAA templates (Figure 7) [58]. These CNTs-NAA membranes were demonstrated to be useful for tuning the transporting of dye molecules. In other recent studies by our group, CVD technique was used to deposit monolayers of organosilane (*i.e.*, APTES and MPTES) inside NAA pores [59,60]. To this end, NAA substrates were heated to temperatures in excess of 110 °C with these silanes under vacuum conditions. APTES modification of NAA substrate were used to selectively bind biomolecules, whereas MPTES (mercapto-silane) modified NAA were used to selectively detect gold and mercury ions in aqueous media.

#### 3.2.2. Wet Chemical Techniques of Surface Modification

Attaching other molecules to the surface of NAA is a productive manner to use its high surface area. The inherent presence of anionic impurities in its structure makes the pore surface of NAA prone to attack by oxides to generate surface hydroxyl groups, which act as nucleation centers for its further covalent functionalization. The advantage of wet chemical approaches is that they are usually based on self-assembly phenomenon, which results in full and uniform monolayer surface coating. Unlike physical surface modification, chemical surface modification result in no noticeable change in the structural properties of the NAA substrate [29]. Several groups of compounds such as carboxylic acids, organosilanes and phosphonic acids have been used to impart selective surface chemistry to NAA surface. These compounds are known to form highly uniform monolayers on NAA surfaces.

##### Self Assembled Monolayers

Self-assembled monolayers (SAMs) are assemblies of organic molecules formed by spontaneous attachment and arrangement of molecules from the liquid phase onto solid surfaces. SAMs of desired molecules can be prepared on NAA in several ways. Pioneering works were focused on self-assembly of alkanethiols on gold-coated NAA surfaces [61]. This process has been extended to SAMs based on organosilanes and phosphonates, which can present a broader range of functional terminals (e.g., amine, carboxyl, epoxy, *etc.*).

##### Organosilane Modification of AAO

**Figure 8.**
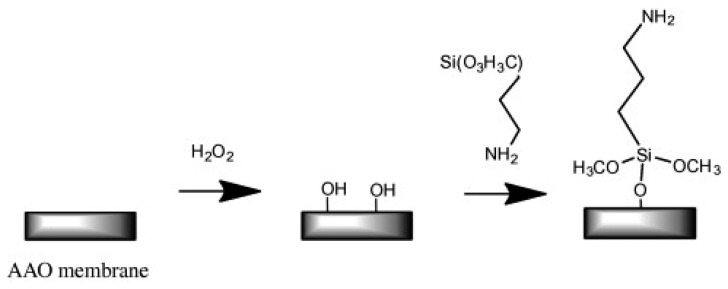
Schematic of silanization process used for modifying NAA. (Adapted with permission from [62]).

Organosilanes can be readily formed onto the native or hydroxylated surface of NAA. Organosilanization of NAA has been effectively used to control its wettability and adsorption properties. Wide varieties of organosilanes are commercially available, which can be covalently bound onto the surface of NAA by simply incubating the hydroxylated NAA substrate in silane solution [62]. A schematic of silanization of hydroxylated NAA is provided in Figure 8.

The variety of silanes used for modifying NAA and their applications are listed in Table 2. Tuning the wettability of NAA surface has been demonstrated by selectively forming SAMs of silanes with hydrophobic terminal groups such as alkyl-trichloro-silanes or perfluoroalkyl-silanes [5,63–65]. Ku *et al.* demonstrated that alkyl-trichlorosilanes with chain length C1–C8 can be used to effectively render the surface of NAA completely hydrophobic, such that even after fully immersing the NAA into an aqueous medium (buffer in this case), pores remain filled with air [65]. In contrast, silanes with active functional groups as PEG-silanes, amine-terminated silanes, epoxy-terminated silanes and others have been used to increase the wettability of NAA. Functionalization of NAA by PEG-silane has been proven to prevent NAA structures from biofouling for long-term use under *in vitro* or *in vivo* conditions, improve biocompatibility for immunoisolation, and to reduce the effective pore diameter of NAA for molecular separation applications [66–68].

Furthermore, SAMs based on silanes have been extensively used as an initial active layer to immobilize other (bio)molecules, polymers, nanoparticles, DNA, cells, quantum dots, and lipid bilayers onto the surface of NAA structures [29,69–72]. Different silane chemistries can be used for immobilizing biomolecules such as growth proteins, peptides, lipid bilayers and antibodies. Amid them, amino-propyltryethoxy silane (APTES) is the most commonly used silane for modification of NAA. APTES modification of NAA was shown to suspend lipid bilayers over the pores by first grafting N-hydroxy-succinimidyl carbonate-polyethylene–glycol (NHS-PEG) and subsequently fusing the vesicle onto the PEG layer [73]. Amine groups in APTES have also been revealed to act as surface-confined initiator for grafting of polymer brushes of poly(γ-benzyl-l-glutamate) (PBLG). PBLG polymer brushes can tailor the filtration and separation properties of NAA membranes [74]. Furthermore, APTES-modified NAA can be used to graft poly-N-isopropylacrylamide (PNIPAM) through atom-transfer radical polymerization (ATRP) [75]. Additionally, APTES-modified NAA supports have been used to assemble and fabricate metal nanoparticles or nanotubes by electroless deposition. Wang *et al.* synthesised Pd nanotubes using APTES-modified NAA templates, which were treated in an aqueous solution of SnCl_2_ and HCl and subsequently immersed in a solution of PdCl_2_ and HCl for specific time to generate Pd nanotubes by electroless deposition approach [76].

Sehayek *et al.* decorated NAA membranes with electrically conductive nanoparticles by passing them through APTES-functionalized NAA pores [78]. Lahav *et al.* also demonstrated the fabrication of bimetallic nanotubes of Au-Pd by immobilizing a mixture of Au and Pd nanoparticles inside APTES-modified NAA. This leaded to solidification of nanoparticles and formation of multiwall metallic nanotubes upon drying [79].

**Figure 9.**
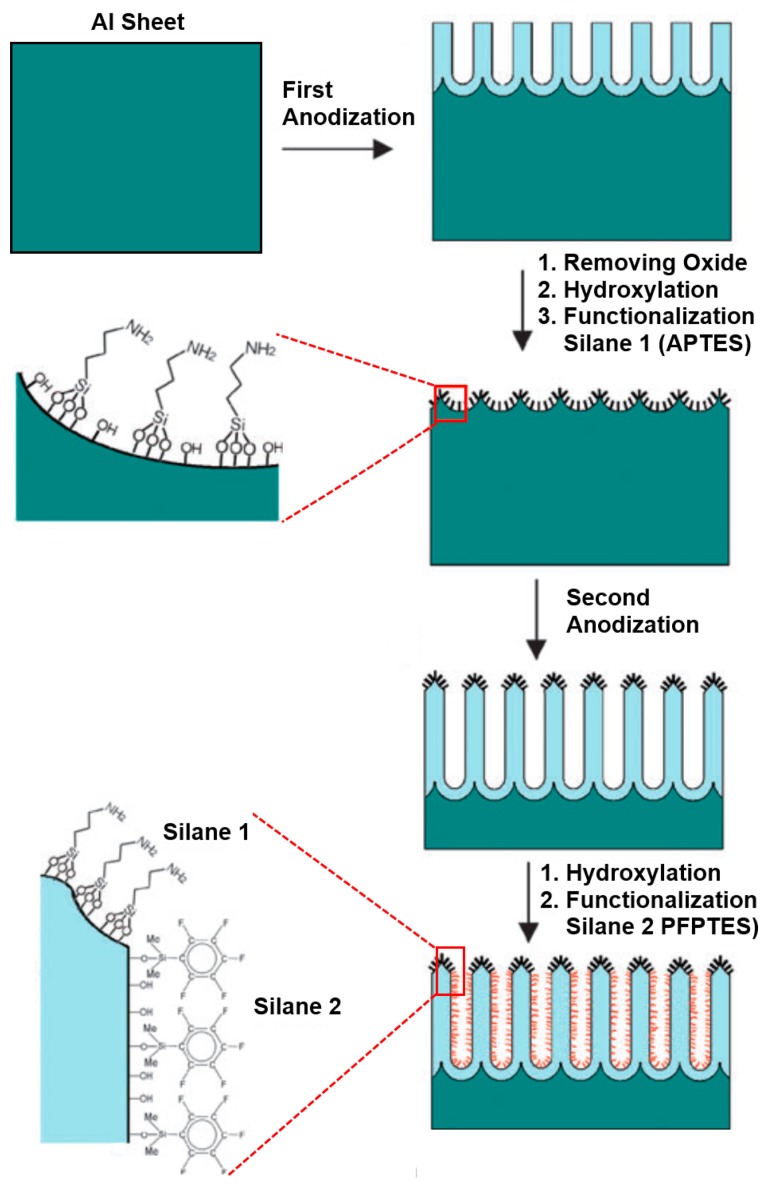
Schematic illustrating of silanization process for functionalizing the surface of NAA with multiple silanes (Adapted with permission from [77]).

Our group demonstrated an innovative approach to attach multifunctional silane-based layers in a single NAA template. This multilayered surface modification approach was achieved by a series of anodization and silanization cycles repeated in a sequential fashion. This approach is useful for applications as membrane filtration and separation, as two different surface chemistries can be obtained with distinctly surface properties [77]. We successfully functionalized the surface of NAA with two or three layers of different silanes (pentafluorophenyl-dimethylpropylchloro-silane (PFPTES), APTES and N-triethoxysilylpropyl-(O-polyethyleneoxide)urethane (PEG-silane)), providing a range of surface functionalities and wettabilities as shown in Figure 9 [77,80]. This approach makes it possible to control the thickness of each functional silane layer by controlling the thickness of NAA grown during each anodization and silanization cycle. We further demonstrated the application of this method by selective transport of hydrophobic and hydrophilic dye molecules through membranes with hydrophobic and hydrophilic layers.

##### Functionalization with Organic and Phosphonic Acids

**Figure 10.**
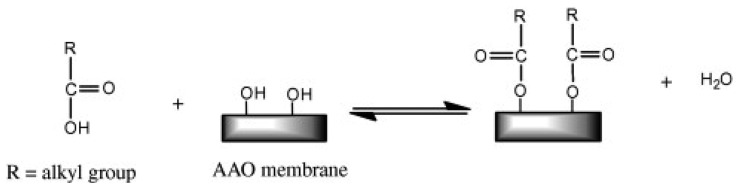
Functionalization path of NAA surfaces with *n*-alkanoic acid. (Adapted with permission from [82]).

Allara and Nuzzo, initially reported on the qualitative and quantitative assessments of self-assembly of *n*-alkanoic acids of varying carbon chains (16–22 carbons) and terminal groups such as methyl, vinyl or propargyl groups [81,82]. Figure 10 defines the basic attachment process of *n*-alkanoic acids to NAA. Chang and Suen, displayed successful functionalization of NAA surface with n-alkannoic acids (*i.e.*, carboxylic acids) using a solvent-based functionalization method [83]. Cheow *et al.* used octanoic and octadecanoic acid as well as fluorinated organic acids such as trifluoroacetic acid, perfluoropentanoicacid and 2,3,4,5,6-pentafluorobenzoic acid, to impart hydrophobicity to NAA surfaces. The highest contact angle achieved by these functionalization approaches was 107° for perfluoropentanoic acid-modified NAA [84]. However, the functionalization process was reversible and the resulting functional monolayers were found to be unstable in aqueous environments. Similarly, Karaman *et al.* also modified the surface of NAA with a variety of short carbon chain (3–4 carbons) fluorinated and non-fluorinated carboxylic acids [85]. Eliasson's group have also shown that other organic molecules such as stearic acid and methyl stearate can form monolayers onto the surface of NAA [86].

Phosphonic acids, on the other hand, do form stable monolayers on NAA surfaces , but there are only few examples of these compounds self-assembling onto NAA [29]. Recently, Debrassi *et al.* provided a comprehensive study on the stability of a range of monolayer forming compounds, including 1-hexadecylphosphonic acid (PA: phosphonic acid), 1-hexadecylcarboxylic acid (CA: carboxylic acid), 2-hydroxyhexadecylcarboxylic acid (2OHCA: α-hydroxycarboxylic acid), 1-hexadecyne (YNE: alkyne), 1-hexadecene (ENE: alkene), and 1-hexadecyltrimethoxysilane (SIL: silane) [87]. NAA modified with phosphonic acids was the most stable over a pH range 4–8 and temperatures up to 80 °C. They also found that the stability of NAA substrate modified with PA depends strongly on the terminal functional group of the monolayer. For instance, hydrophobic layers were less susceptible to pH and temperature changes than hydrophilic ones. In recent studies by our group, water soluble phosphonic acid (*i.e.*, 2-carboxyethyl phosphonic acid) was used to further attach streptavidin onto the surface of NAA [88,89]. These streptavidin-modified NAA substrates were then employed for impedance-based sensing of biotin (analyte) in aqueous buffer solution.

##### Lipid Bilayers

Lipid-bilayers are known to mimic cell membranes to a great extent, although their poor stability hinders their applications in several fields. NAA is accepted as a suitable substrate for supporting lipid-bilayers as different assemblies of lipid-bilayers can be obtained depending on the structural features of NAA and deposition methods with the ability to suspend lipid-bilayer on the NAA membrane top or confine them inside the nanopores [90,91]. Suspended lipid-bilayers on NAA substrates have shown long-term stability and act as channels for transporting ions. These suspended lipid-bilayers were fabricated by fusing lipid vesicles on gold-coated NAA substrates pre-treated to form SAMs of alkanethiols with negatively charged head group. Their fabrication approach consisted of gold coating of NAA surface followed by formation of a SAM of alkanethiols with a negatively charged head group [90,92,93]. Similarly, confined lipid-bilayers were fabricated using silanes-coated NAA membranes to achieve fusion of lipid vesicles inside the nanopores [94]. Smirnov and Poluektov, used this technique to deposit multiple lipid-bilayers and obtain lipid nanotubes [95]. They were also able to control the thickness of the lipid wall and confirmed the formation of lipid nanotubes through electron paramagnetic resonance, nuclear magnetic resonance spectroscopy and fluorescence microscopy.

**Figure 11.**
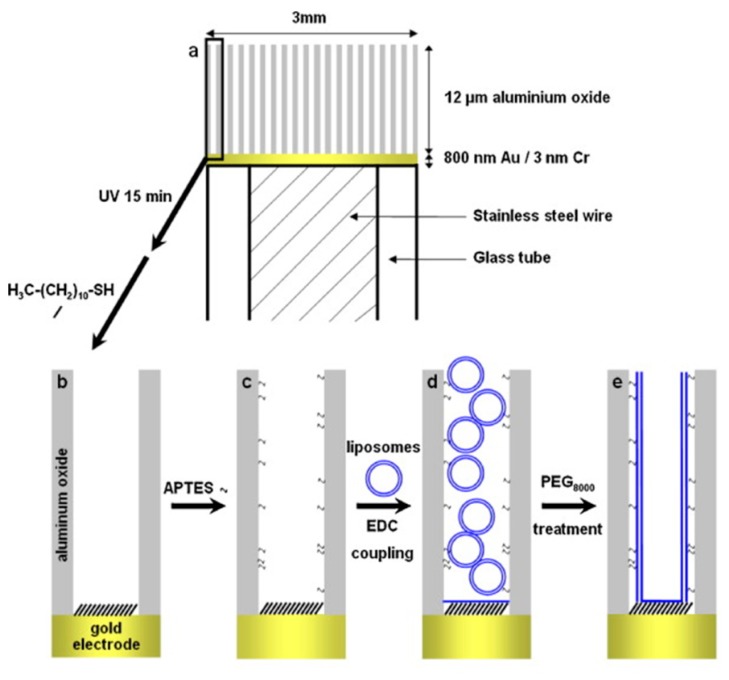
Schematic presenting the step-wise process used to fabricate lipid-bilayers inside NAA pores using PEG triggering. (Adapted with permission from [73]).

Largueze *et al.* used a PEG-triggered fusion of lipid vesicles to deposit tethered lipid bilayers inside the pores of NAA [73]. To this end, NAA pores were functionalized with APTES and one side of the NAA membrane with a SAM of undecanethiol after gold coating. Then these multifunctional NAA membranes were incubated with lipid vesicles. After a fixed period of time these lipid vesicles were fused to NAA pore walls using PEG as a trigger (Figure 11). The potential of fabricating lipid-bilayers lies in designing sensitive biomimetic nano-channels that can be used for ion transporting and sensing applications.

##### Layer-by-Layer Deposition

**Figure 12.**
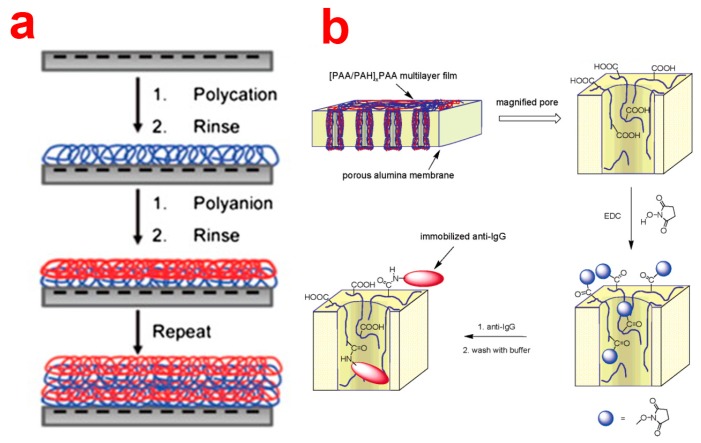
(**a**) A general scheme showing steps for layer-by-layer deposition of polyelectrolytes; (**b**) LbL modification of NAA pores used to immobilize antibodies. (Adapted with permission from [96] and [101], respectively).

Layer-by-Layer (LbL) deposition is a very simple, inexpensive, and versatile technique used to obtain thin polyelectrolyte (PE) multilayers, the thickness of which can be controlled with nanometric resolutions, on different substrates. This is achieved by alternatively dipping the substrate into polyelectrolyte solutions of opposite charge (Figure 12a) [96]. This technique was demonstrated to be able to deposit PE layers inside NAA pores. So far, LbL technique has mainly been used to tune the transport properties of NAA membranes, attach nanoparticles or biomolecules inside NAA pores, and fabricate polyelectrolytic nanotubular structures using NAA as a template [29]. In this regard, Balachandra *et al.* used UV/ozone-treated NAA to adsorb Cu^2+^ ions, which were used to deposit multi-layers of PAA and PAH inside NAA membranes [97]. Notice that NAA pores remain open (or not blocked) after deposition of up to seven PE bilayers. So far, a vast number of PE combinations and compositions (e.g., PSS/PAH, PSS/PAH, (PSS/PDADMAC)_4_PSS) have been used to efficiently control the flux, transport, rejection rate and selectivity of solutes through LbL-functionalized NAA membranes [98–100]. Furthermore, Dai *et al.* used LbL technique to immobilize antibodies inside the nanopores of NAA [101]. In this way, antibodies were immobilized onto the deposited layers of PE composed of [PAA/PAH]_3_PAH using carbodiimide coupling chemistry (Figure 12b). The same group showed that positively charged PE layers can be utilized to attach citrate-stabilized gold nanoparticles for catalytic applications [102]. LbL technique has employed NAA to synthesize nanotubes of soft materials such as polyelectrolyte polymers, biomolecules, and metal organodiphosphonates. As an example, Li *and coworkers* introduced the concept of LbL assembly of polyelectrolyte multilayers inside nanoporous alumina template [103,104]. The assembly of polyelectrolyte milt-layers results in polymeric nanotubular structure that display complex but well-controlled wall morphologies and adjustable wall thickness.

They deposited polyallylamine hydrochloride (PAH)/sodium poly(styrene sulfonate) (PSS) from aqueous solutions inside the pores of a NAA membrane assisted by pressure. The alumina walls were subsequently etched using aqueous NaOH solution to obtain liberated flexible (PAH/PSS)_3_ nanotubes, which replicated the length and outer diameter of the NAA templates used in the synthesis process [103]. Similarly, Martin and co-workers reported the synthesis of DNA nanotubes supported by 1,10-decanediylbis(phosphonic acid) skin using sequential deposition of complementary nucleotides with specific sequences in a NAA membrane [105]. In this process, NAA was first immersed into a solution of (DOP) followed by a solution of ZrOCl_2_ to form the (DOP)/Zr(IV) supporting skin. The inner surface of the nanotubes resulted from hybridization between multiple double-stranded DNA layers. The same group also demonstrated, for the first time, the synthesis of protein nanotubes by alternately exposing the NAA template to a solution of the glucose oxidase (GOx) or hemoglobin (Hb) [106]. Finally, glutaraldehyde was used as a cross-linker to hold these proteins together. This study also evidenced that GOx nanotubes catalyzed glucose oxidation, whereas heme-electroactivity of Hb nanotubes was retained. Apart from fabrication of novel nanomaterials, LbL has been used to attach functional molecules inside the pores of NAA. For example, Masumoto *et al.* adsorbed positively charged polyelectrolyte polymer poly-L-lysine inside the pores of NAA to obtain positively charged inner surfaces suitable to bind DNA molecules by electrostatic interactions [107]. A similar strategy has been used to attach noble metal nanoparticles/cubes inside NAA pores (e.g., adsorption of poly(diallyldimethylammonium chloride) to attach gold nanoparticles or PAH/PSS polyelectrolyte multilayers for attachment silver nanocubes) [108].

##### Sol-Gel Chemistry

**Figure 13.**
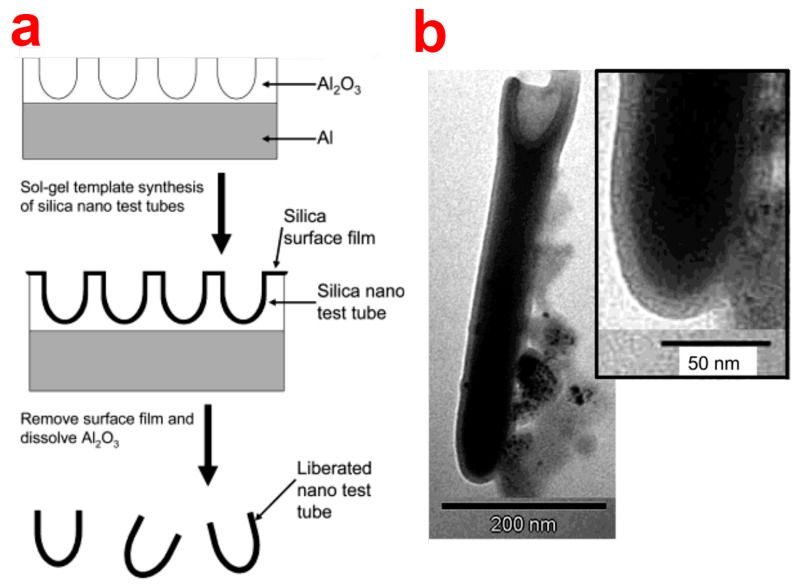
(**a**) A schematic process for templating NAA structure to replicate silica nanotubes via sol-gel process; (**b**) TEM images of the resulting silica nanotubes templated from NAA. (Adapted with permission from [109]).

Sol-gel assembly process combined with NAA templates has been predominantly used to replicate and prepare novel nanostructures as nanotubes and nanorods or reducing the pore diameter and imparting different surface chemistries to NAA structures (Figure 13) [109]. The most widespread materials for sol-gel synthesis of nanostructures using NAA templates include silica, carbon, metal oxides (e.g., TiO_2_, TiO_2_–silica composite, SnO_2_, NiO, *etc.*), and polymer-derived SiOC [29]. This technique is based on the hydrolysis of the sol-precursor on the NAA substrate by immersion, dipping or spin coating followed by solvent evaporation and formation of a glassy gel inside the pores [110]. The remaining solvent from the gel-NAA system is removed by subsequent thermal treatment. Sol–gel technique results in synthesis of high purity homogenous and multi-component structures with the ability to further control their properties such as structure, thermal stability and surface reactivity (using precursors with additional functional groups) [111,112]. Lee *et al.* prepared modified NAA membranes by depositing silica nanotubes using sol-gel template technique [113]. The prepared silica nanotubes were utilized for selective binding and delivery of enantiomeric therapeutic drug. He *et al.* have demonstrated the fabrication of silica nanotubes with different shapes using funnel-type NAA templates [114–116]. The obtained silica nanotubes with increasing diameter from one end to the other. These nanotubes are called as shape-coded silica nanotubes (SNTs) and were used for specific detection of rabbit anti-IgG from a mixture containing rabbit and human IgG antibodies. They also demonstrated that SNTs can be used for multiple analytes at the same time. As a proof of concept, SNTs featuring three different shape codes were functionalized with rabbit, mouse, and human IgG antibodies, which were subsequently used for simultaneous detection of anti-rabbit and anti-mouse IgG antibodies using a sandwich assay.

Wang *et al.* prepared carbon-based nanostructures such as carbon fibers and carbon ribbons with a circular mesoporous framework [117]. Phenol-formaldehyde presol was used as a precursor solution along with surfactants such as F127 (EO_106_PO_70_EO_106_) and P123. First, the precursor solution was infiltrated inside the pores of NAA to form gel during the ageing time followed by calcination at 600 °C for 3 h to produce mesoporous carbon nanostructures. The same group prepared mesoporous titania nanotubes within NAA membranes by replacing the carbon source with a titania source (*i.e.*, titanium tetraisopropoxide, TTIP) and employed the mesoporous titania nanotubes as an electrode material in a high rate rechargeable lithium battery [118]. Platschek *et al.* extended this work by controlling the morphology of synthesized mesoporous carbons by manipulating the sol-to-surfactant ratio and the types of surfactants [119]. Confalonieri *et al.* displayed deliberate control over the spatial arrangement of nanoparticles using a novel method to assist the self-assembly of magnetic nanoparticles. For this, they spin coated iron oxide nanoparticle (20 nm) on the pretextured Al obtained after removal of first anodized NAA layer. The magnetic nanoparticles were found to form clusters of different arrangements within the valleys of pretextured Al, such as collars, chains and hexagonally closed islands. They used memory effect to probe the strength of magnetic interactions between particles [120].

##### Electrochemical and Electroless Deposition of Metals

Electrochemical deposition is a simple, inexpensive method for modifying the pores of NAA with metal nanostructures. In comparison to other metal deposition techniques such as CVD, ALD or thermal metal deposition, the electrochemical method does not require expensive equipments and special conditions [121]. Different metal-based nanostructures (*i.e.*, nanowires, nanorods, nanotubes and nanoparticles) have been templated from NAA using this approach [29]. Recent developments in electrochemical deposition have made it possible to deposit multiple metals in the same NAA template for producing alloy nanostructures. Liu *et al.* prepared segmented Ag-Au alloy nanowires inside NAA templates using electrochemical deposition [122]. Burdick *et al.* demonstrated the fabrication of multisegmented nanowires of gold with short Ag spacers [123]. Generally, electrochemical deposition is carried out on NAA membranes, which makes it difficult to deposit metal inside thin NAA membranes. To overcome this, Lee *et al.* developed an alternating current (AC)-based electrochemical deposition technique to assembly gold nanoparticles within NAA membranes [124]. Recent advancement in electropolymerization have lead to fabrication of multisegmented composite nanorods and nanowires of conducting polymers with metals and semiconductors prepared by electrochemical deposition [121]. These metal decorated NAA substrates have been recently used as sensing platforms for such optical techniques as surface enhanced Raman spectroscopy. This was explored by Masuda's Group, who created a 3-D ordered mosaic of gold nanoparticles using the pores of NAA as a mask. The resulting nanostructures were used as sensing platforms in SERS-based detection of pyridine [125]. Electroless deposition, on the other hand, involves reduction of metal cations from an aqueous solution [126]. Martin and co-workers used this method to prepare gold/NAA composites, which were investigated for molecular separation processes [61]. Either metal nanotubes or nanorods/wires can be obtained from electroless deposition depending on the deposition time. So, nanotubes are obtained with short times while solid nanorods/wires can be produced by longer deposition times [121]. Mu *et al.* fabricated SERS active arrays of Au nanoparticles with tunable particle gaps on NAA substrates. The pH and temperature conditions of the plating bath were reported as the main factor for controlling the size, shape and aggregation of Au nanoparticles during the electroless deposition process [127]. Wang *et al.* prepared a number of metal nanotubes (*i.e.*, Co, Ni and Cu) using NAA templates via electroless deposition on APTES-modified NAA membranes [76]. The inner diameter and length of the resulting nanotubes were adjusted by controlling the deposition times and the thickness of the NAA template, respectively. Other investigations synthesized Ag nanotubes with lengths over 10 μm inside NAA membranes. Furthermore, Ag nanoparticles of controlled size and smooth surface have been deposited inside NAA using this technique [29].

**Figure 14.**
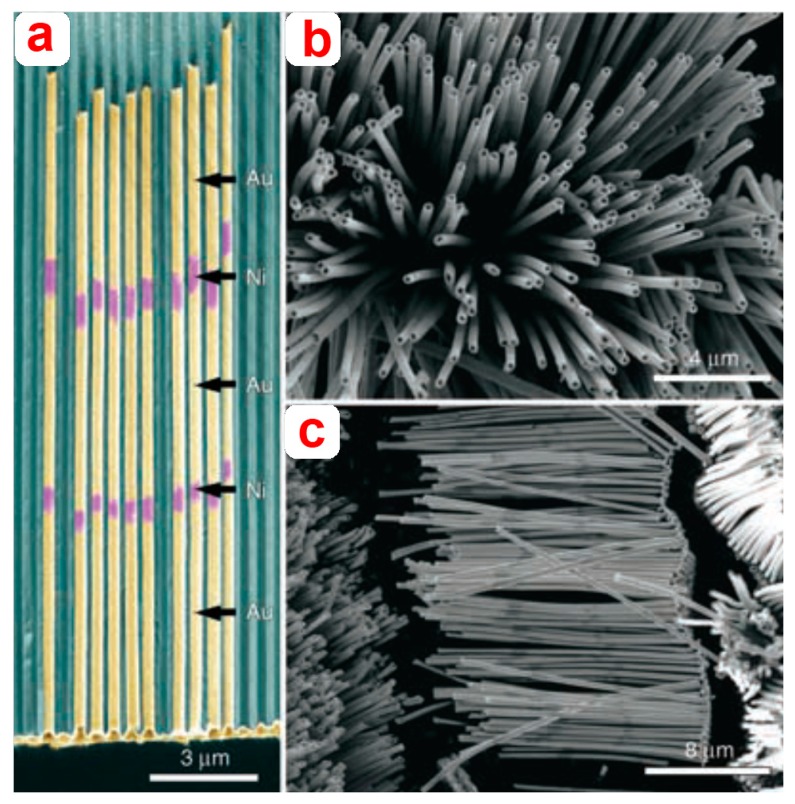
SEM images of multisegmented metal nanotubes with a stacked configuration of metal inside NAA templates. (**a**) Cross-sectional SEM image of Au-Ni-Au-Ni-Au along the nanotube axis (Au: yellow and Ni: purple) (**b**) and (c) SEM images of multisegmented metal nanotubes after dissolution of the NAA in NaOH. (Adapted with permission from [128]).

Lee *et al.* fabricated multisegmented metallic nanotubes by combining electroless and electrochemical deposition methods (Figure 14). In this approach, Sn (II) was first adsorbed onto the pore walls of NAA membranes from a solution of SnCl_2_. Subsequently, exposure to Ag (I) led to spontaneous reduction and replacement of Sn (II) via a sensitization process, which was repeated several times. Ag nanoparticles decorated inside NAA membrane pores were isolated from each other, thus did not conduct current. Final electrochemical deposition of Au resulted in formation of Au nanotubes with a bimetallic stacked configuration [128]. Zhang *et al.* used AC electrodiposition to fabricate nanoporous anodic alumina films embedded with Fe. These films display vivid structural colors and magnetic properties. These properties were found to be dependent on oxidation time of aluminum [129].

## 4. Structural Engineering and Optical Optimization of NAA

**Figure 15.**
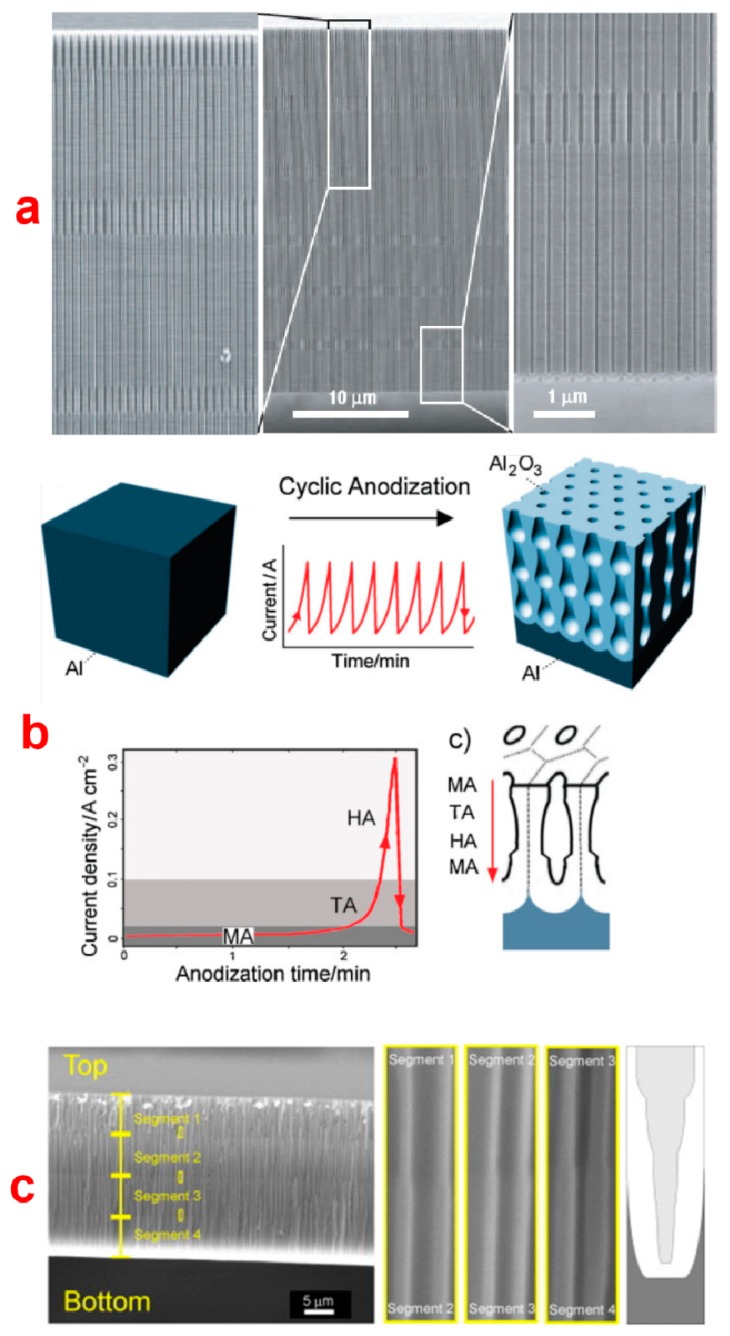
(**a**) Cross-sectional SEM images of pore diameter modulations in NAA produced by switching the anodization between MA and HA regimes; (**b**) Schematic illustration of cyclic anodization process introduced by Losic *et al.*; (**c**) Cross-sectional SEM images of high aspect ratio funnel-like NAA produced by Santos *et al.* (Adapted with permission from [8,24,25]).

The porous architecture of NAA plays a key role in defining its optical properties. The nanoporous structure of NAA can be designed and engineered to produce a broad range of pore geometries with tailored optical properties. Engineered NAA structures can be designed to interact with light in different manners as to confine, guide, transmit, emit, and reflect it. Developments of different electrochemical anodization approaches have made it possible to design and engineer the structure of NAA to produce a new generation of optical nanostructures. So far, a large variety of NAA pore geometries (*i.e.*, modulated, hierarchical, serrated, three-dimensional, funnel-like, and multilayered) have been engineered by using innovative and unique anodization techniques [130–132]. Figure 15 shows SEM images and schematics of some of the most representative NAA structures generated by different electrochemical approaches. The precise engineering of the nanoporous structure of NAA can result in the generation of optically active structures such as distributed Bragg reflectors, microcavities, rugate filters, omnidirectional mirrors and waveguides [132]. These optical nanostructured devices are formed due to the ability of electrochemical anodization process to produce variations in porosity (*i.e.*, refractive index) of the NAA structure depending upon the type and regime of anodization. The most commonly used method to produce porosity variations is to switch between “Hard” and “Mild” anodization regimes repeatedly, which present different levels of porosity (*i.e.*, MA results in 10% porosity while HA only produces NAA with 3% porosity). Lee *et al.* used this approach to fabricate NAA with pore diameter modulations along the length of the pores (Figure 15a) [8]. In this method, perfectly ordered NAA was first prepared on an electropolished Al substrate in phosphoric acid electrolyte (H_3_PO_4_: 0.4 M, 110 V and 10 °C) after imprinting the pattern from a master stamp. After 15 min of anodization in H_3_PO_4_ the electrolyte was replaced with 0.015 M oxalic acid (H_2_C_2_O_4_) and further anodized at 137 V at 0.5 °C for 2 min. NAA with highly uniform periodic modulations of the pore diameter and constant inter-pore distance were obtained by repeating the aforementioned two anodization processes. Notice that the anodization voltages were set such that they yield the same inter-pore distance in MA and HA conditions and prevented NAA pores from pore branching. The pore diameter of NAA during each anodization step was controlled by the anodization voltage and the acid electrolyte, whereas the length was controlled by the anodization time. This report inspired and let to overwhelming a number of studies on different electrochemical approaches to generate pore diameter modulations in NAA structures. As for this, Pitzschel *et al.* fabricated similar pore diameter modulations in NAA using the same electrolytes under different anodization conditions. In this case, the period of nanoimprint from a master stamp was 235 nm. They performed the first MA step under 72 V in 0.4 M aqueous H_3_PO_4_ at 10 °C and the hard step was performed under 93 V in 0.15 M H_2_C_2_O_4_ at 5 °C in a 4:1 water/ethanol mixture. They further used this pore modulated structure to synthesize magnetic nanotubes by depositing Fe_3_O_4_ using ferrocene and ozone as precursors [53]. Losic *et al.* on the other hand, used a cyclic anodization approach, where pore modulations along the length are obtained by applying periodic oscillatory current signals with different profiles, amplitudes, and periods (Figure 15b**)** [24].

Santos *et al.* showed the fabrication of hierarchical type NAA where first anodization step is to obtain pre-patterned Al chip with concave cavities [133]. Subsequently, these pre-patterned Al chips are anodized under asymmetric anodization conditions (*i.e.*, changing one or more than one anodization conditions than the first anodization). The resulting NAA structure was observed to have multiple pores within single concave cavities, thus called a hierarchical NAA structure. Serrated-type NAA pores were first fabricated by Zhu *et al.* They also proposed a new growth model for NAA, which emphasizes on the close relationship between pore generation and oxygen evolution during the formation of such NAA structures [134]. This study was further extended by Li *et al.* who also on the basis of the results obtained from their experiments and simulation attributed the initiation and formation of serrated-like NAA pores to the evolution of oxygen gas bubbles during the anodization [135]. 3-D type nanoporous structure in NAA was obtained by Losic *et al.* after combining cyclic anodization and wet chemical etching using phosphoric acid [28]. Likewise, Santos *et al.* combined discontinuous anodization and wet chemical etching steps with phosphoric acid to obtain a similar 3-D NAA pore structure [136].

Another interesting structure based on NAA is the funnel-like NAA, which can be produced by combining multiple anodization and pore widening steps [24,137,138]. This structure features a stack of NAA layers of decreasing pore diameter from top to bottom, therefore called funnel-like NAA structure (Figure 15c) [23]. The length of each stack is controlled by the time of anodization or the total charge (*i.e.*, integrated current passed through the system), whereas the pore diameter in that stack is established by the pore widening time. Nagaura *et al.* fabricated low aspect ratio funnel-like NAA and studied their structural changes by manipulating the number of anodization and pore widening cycles [137]. The same authors replicated this low aspect ratio funnel-like structure to create nickel films with nanoconical surface morphology [138]. Yanagishita *et al.* applied similar approach and replicated the funnel-like structure featuring different pore shapes to a polymer by photo-imprinting process [139]. The polymer replicates were studied for their antireflection behavior by measuring the amount of transmitted light. NAA nanostructures with more gradual changes in slope were found to be less reflective. Santos *et al.* fabricated high aspect ratio funnel-like NAA by controlling the length of each stack very precisely according to the total charge passed through the system. They demonstrated NAA funnel-like structure with two, three, and four stacks [24]. Li *et al.* reported tailoring of the shape of funnel-like NAA pore structures by new electrochemical approach [140]. They successfully demonstrated the fabrication of linear cones, whorl-embedded cones, funnels, pencils, parabolas and trumpets-like nanopore structures by controlling the fabrication parameters as anodization time, etching time and cycle times. Very recently, we reported on the fabrication of inverted funnel-like NAA, which has increasing pore diameter along the pore length from top to bottom [32]. This innovative structure was achieved by taking advantage of the fact that the chemical dissolution rate of NAA decreases after annealing at temperatures in excess of 100 °C. We made inverted funnel-like NAA with two and three stacks by combining annealing steps and anodization steps. In addition, formation of these inverted funnel-like structures was monitored in real-time using reflective interference spectroscopy method.Multilayered NAA structures can be used to fabricate a broad range of optical nanostructures such as DBRs, optical microcavities, rugate filters and other optical and photonic structures. They consist of multiple layers of NAA segments with different levels of porosity. Multilayered NAA structures have been fabricated by periodically alternating the voltage during the anodization process. Anodization profiles such as stepwise, sinusoidal, pseudo-sinusoidal, saw-like, *etc.* have been applied to engineer the porosity (*i.e.*, refractive index) of NAA in depth. This allows for specifically designing and engineering the light-matter interaction in NAA for subsequent applications in chemical and bio-sensing. Lee *et al.*, for the first time, displayed the ability to fabricate multilayered NAA via an electrochemical approach consisting of periodic pulses of low and high potential (*i.e.*, voltage in MA and HA regime) [26]. In this study, they performed anodization of pre-patterned Al chips through pulse anodization under potentiostatic conditions in sulphuric and oxalic acids. Nanotubular array structures with periodic neck-like constrictions along the length of the tubes were observed for sulphuric acid electrolyte while NAA with periodically varying pore diameter were observed for oxalic acid electrolyte.

Sulka *et al.* applied a similar approach to fabricate layered NAA structures, which were subsequently used as templates for fabricating modulated metal nanowires. In another study, they demonstrated the application of this multilayered NAA structure as distributed Bragg reflector (DBR) [141]. The resulting NAA-based DBR structure had periodically modulated layers of NAA with low and high porosity (*i.e.*, refractive index). These DBR mirrors were observed to effectively reflect light in two different ranges of wavelength, which was in good agreement with the mathematical calculated reflection spectrum.

Zheng *et al.* prepared NAA based DBRs by anodizing Al chips under pseudo-sinusoidal voltage profile in oxalic acid electrolyte [142]. They studied the effect of the anodization temperature on the transmission peak of the prepared DBRs. The results predicted that almost the whole range of visible spectrum can be covered by adjusting the anodization temperature between 7 and 14 °C. Recently, Rahman *et al.* used an innovative cyclic anodization approach produce NAA-based DBRs [143]. They also studied the effect of pore widening time on the position of the photonic stop-band of the resulting DBRs. This study demonstrated the ability to control the photonic stop-band of these optical nanostructures by modulating the refractive index contrast between the layers through pore widening. More recently, Kumeria *et al.* fabricated nanoporous rugate filters by anodizing Al chips under pseudo-sinusoidal potentiostatic conditions in oxalic acid electrolyte [132]. They prepared four different types of rugate filters based on NAA and selected the most optimum structure based on their experimental and theoretical modeling (*i.e.*, the Looyenga−Landau−Lifshitz model) for sensing applications. This study demonstrated that optimize optical signals enable the precise control of the optical properties of nanoporous structures for chemical and bio-sensing applications.

## 5. Optical Sensing Applications

### 5.1. Plasmon Resonance Sensors Based on NAA

**Figure 16.**
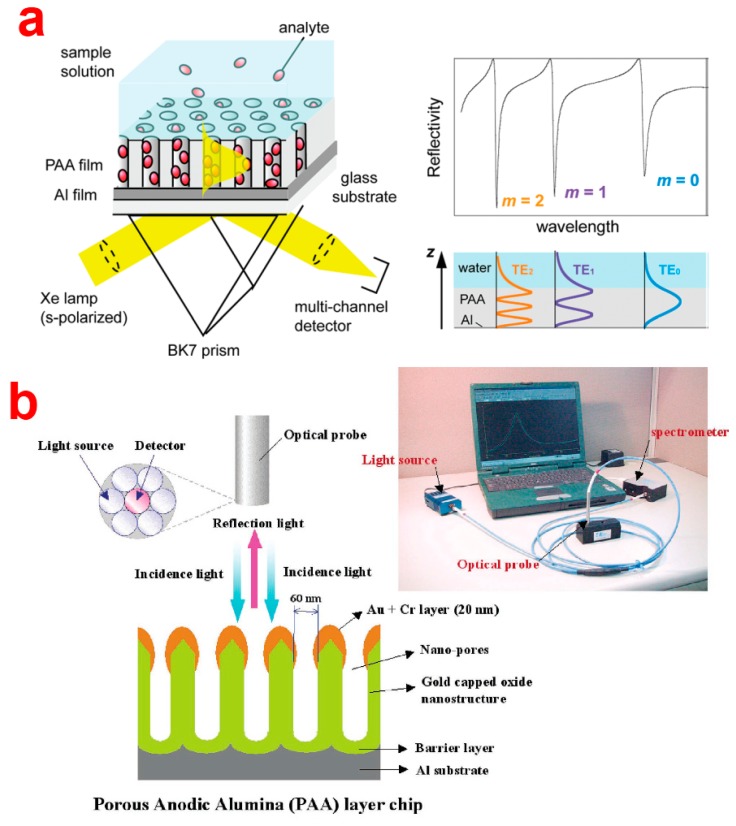
(**a**) Schematic illustration of the SPR-NAA waveguide sensor developed by Hotta *et al.* and its typical reflection spectrum; (**b**) Schematic of NAA-LPR setup developed by Kim *et al.* by coating the top surface of NAA with gold to form nano-caps. (Adapted with permission from [150,151], respectively).

Since the first publication in the late 1970s, the potential of surface plasmon resonance (SPR) for characterization of thin films and monitoring analyte-receptor binding processes at metal interfaces has been greatly recognized [144]. Capabilities of SPR are real-time and in-situ measurements of a wide range of surface interactions, ligand-binding affinity, association/ dissociation kinetics, affinity constants, and highly sensitive surface-concentration measurements. During the last decades, SPR has been exploited such that more than 75% of research on optical biosensing was based on SPR [144]. This extensive exploitation of this sensing technique has resulted in the generation of new SPR configurations (*i.e.*, Grating, Otto, and Kretschmann configurations) and substrates (*i.e.*, nanostructured films, porous materials, nanoparticles, *etc.*). The use of nanomaterials has led to the discovery of a new type of SPR such as waveguiding and localized SPR (LSPR or LPR) that exclusively occurs in noble metal nanoparticles/structures. Notably, when the noble metal particles/structures (*i.e.*, gold, silver, *etc.*) are scaled down to nanometric level (*i.e.*, <100 nm), light interacts with particles much smaller than the incident wavelength [144]. This leads to the generation of plasmonic oscillations of a particular frequency that are localized around the nanoparticles. LPR oscillations are dependent on the size, shape and surrounding environment of the nanoparticles/structures. The LPR is sensitive to changes in the local refractive index environment, which can be measured through shifts in the resonance wavelength or resolving the LPR. Recent studies have proven that close organization of metal nanoparticles/structures even leads to enhancement of LPR signals. In recent years, NAA has become a very attractive nanomaterial as a substrate for SPR-based sensing devices in SPR, waveguiding (WG), as well as LPR mode [145–147]. In this regard, typically a thin layer of NAA is either directly grown onto a prism or pre-formed NAA is glued to a metal coated prism using a refractive index matching glue. Surface plasmons are excited by an evanescent electromagnetic wave produced by the incidence of light on the surface under a Kretschmann configuration. In addition, NAA coated with noble metals presents highly organized metallic nano-caps, which have been utilized to excite plasmons under LPR mode and applied for chemical and bio-sensing applications. NAA-LPR-based SPR sensors are simple, inexpensive, compact and portable as it does not require a prism-based setup. Notice that the plasmonic characteristics of the SPR devices depend mainly on the surrounding refractive index within a range of few hundreds of nanometers beyond the metal film in Kretschmann configurations and LPR mode [144]. Thus, a thin layer of NAA within this thickness range can be applied onto the prism and used to develop nanoporous SPR sensors. Lau *et al.* combined NAA with waveguiding to develop a WG optical sensing platform, which was used to monitor reversible adsorption and desorption of bovine serum albumin (BSA) at different values of pH [146]. Their NAA-WG setup was based on a Kretschmann configuration with an intermediate Au layer between the prism and the NAA platform. A simpler and sensitive setup was developed by Yamaguchi *et al.* by directly depositing Al onto a prism and anodizing it to generate a nanoporous layer of 200 nm thick. The remaining unanodized Al (17 nm) acted as the metal layer to excite the plasmon for waveguide coupling [148]. Koutsioubas *et al.* on the other hand, fabricated NAA-based SPR sensors used to detect the formation of SAMs of octadecyl-phosphonic acid (ODP) [145]. Dhathathreyan demonstrated that the similar system can be used to quantify enzyme kinetics reaction [149]. In this study, the enzyme invertase was immobilized in the pores of 3 μm thick NAA layer with a pore diameter of 60 nm. The enzyme-substrate was flowed through a custom made flow cell and the digested product was detected by SPR. This system made it possible to study the activity of the immobilized enzyme, which was determined for different concentrations of sucrose at pH ranging from 3 to 6.5. The SPR results revealed a biphasic kinetics for both the adsorption of the enzyme and its interaction with the substrate. Maximum enzyme activity was observed at pH 4.5. In another study, Lau *et al.* monitored the grafting of poly(g-benzyl-L-glutamate) (PBLG) within the nanopores of NAA in real-teal time and in-situ using a SPR-NAA system [74]. Their results reveal that the conformation of PBLG inside the nanopores of NAA is due to confinement of polymer chains inside the pores, which was confirmed by comparing the results obtained with NAA and planar silicon dioxide surface. More recently, Hotta *et al.* used NAA-WG configuration demonstrated by Yamaguchi *et al.* and studied the enhancement of sensitivity (Figure 16a) [150].

For this, the structure of NAA (*i.e.*, waveguiding layer) on Al (*i.e.*, cladding layer) was carefully engineered in terms of porosity, pore density, thickness, and refractive index to tune and optimize the optical WG and enhance the sensitivity of the system. The performance of the optimized NAA-WG was assessed by monitoring the adsorption of BSA molecules onto the nanopores of NAA. The most optimum NAA-WG sensor resulted in an extraordinarily large red shift (>300 nm) of a waveguide mode on adsorption of BSA. This extraordinary red shift was attributed to the large surface area of NAA, which results in a huge adsorption capacity. The theoretical calculations using Fresnel's equation suggested that the sensitivity of the NAA-WG sensor was much higher than that of conventional SPR sensors.

Another study by the same group, reports on enhancement of fluorescence signal on adsorption of fluorophore-labeled BSA (*i.e.*, BSA-AF) inside the nanopore surface of a NAA waveguiding film [152]. They monitored the adsorption of BSA-AF simultaneously using SPR and fluorescence spectroscopy. Enhancement of FL signal on NAA-WG is attributed to a combination of large surface area of NAA and enhanced field due to waveguiding modes within the pores of NAA. As described above, LPR is another type of widely used SPR mode in combination with NAA substrate coated with noble metals (mostly gold or silver). This results in the formation of highly ordered arrays of metallic NPs of gold or silver (*i.e.*, nanocaps) on the top surface of the NAA. Figure 16b shows a typical LPR-NAA label-free biosensor fabricated on a gold-capped AAO substrate for detection of antigen-antibody binding. So far, NAA-based LPR sensors have been demonstrated for specific detection of proteins (e.g., BSA, avidin, DNA and thrombin) or binding events between biotin-avidin, complementary oligonucleotides, and 5-fluorouracil-anti-5- fluorouracil. In this regard, Kim *et al.* developed a new optical biosensor based on a gold-deposited NAA chip [151]. They observed that gold nanocaps resulted in an optical LPR pattern that was highly sensitive to the changes in the effective refractive index of the biomolecular layer attaching them. They demonstrated the detection of up to picomolar quantities of un-labeled oligonucleotides and the hybridization with synthetic DNA samples. Hiep *et al.* deposited gold on the top surface of NAA to form nanocaps and developed a NAA-LPR sensor to detect specific interactions between biomolecules such as biotin and avidin and 5-fluorouracil and anti-5-fluorouracil [147]. Another studies from the same group utilized NAA as a support for plasmonic gold nanoparticles (AuNPs). NAA fabricated by the two-step anodization process was exposed to cationic poly(allyl amine) to attach AuNPs on its surface. The pores of NAA trapped Au nanoparticles. Their experimental and simulation results show that the plasmonic spectra of this NAA-AuNPs composite were enhanced by the interference spectra of the NAA layer. This assembly of AuNPs on NAA substrate showed a high sensitivity towards solutions with different refractive indices [153]. In another study, Hiep *et al.* combined NAA-LPR with an electrochemical system for sensitive detection of toxic peptide (*i.e.*, melittin, the venom from the honey bees) with a lower limit of detection (LOD) at 10 ng/mL [154]. Yeom *et al.* used a similar strategy to form gold nanocaps on the top surface of NAA to develop an immunosensor featuring anti-CRP (C-reactive protein, a cardiac and inflammatory biomarker) antibody immobilised on the gold nanocaps [155]. Their system displayed an extraordinary lower limit of detection for CRP antigen at concentration 1 fg/mL due to LPR enhancement of the optical signals from NAA. Kim *et al.* used a similar system to detect picomolar quantities of untagged oligonucleotides on NAA-LPR platform.

### 5.2. Reflectometric Interference Spectroscopy (RIfS) Sensors Based on NAA

**Figure 17.**
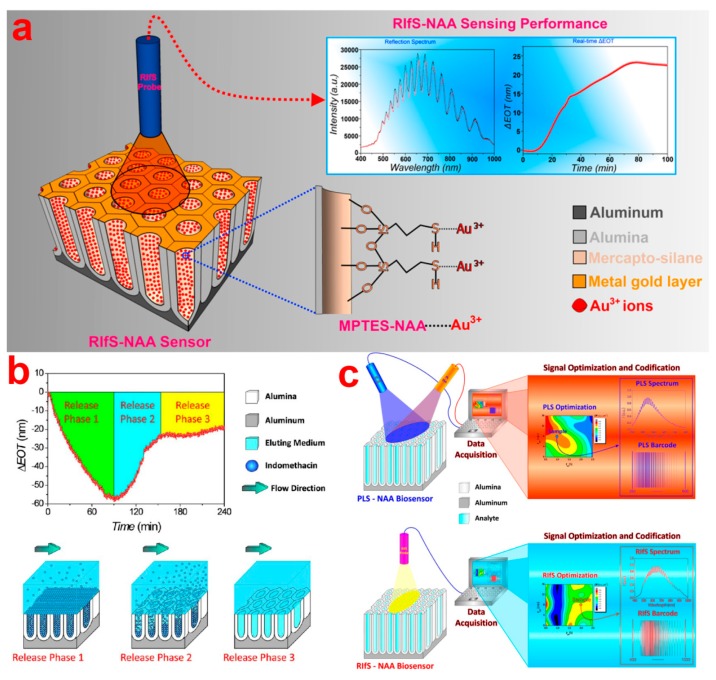
(**a**) A schematic of NAA-RIfS sensor for detection of Au(III) ions developed by Kumeria *et al.* and plot showing its ability for real-time detection; (**b**) Real-time monitoring of drug release from NAA using RIfS; (**c**) The comparative study between RIfS-NAA and PLS-NAA sensors by Santos *et al.* showing ability of the two systems to generate barcodes. (Adapted with permission from [59,60,168]).

Reflectometric interference spectroscopy (RIfS) is another highly sensitive optical detection method that is based on the interaction of white light with films at micrometer scale. In this technique, white light is shined on a thin film and reflected at the two interfaces of the thin film resulting in amplification of the reflected light signal at wavelengths corresponding to the optical modes of the Fabry–Pérot (FP) cavity formed by the system thin film–surrounding medium (*i.e.*, the Fabry–Pérot effect) [156]. A model NAA-based reflective interference along with RIfS spectrum and real-time effective optical thickness change curve is presented in Figure 17a. The wavelength of each interference maximum in the RIfS spectrum is governed by the Fabry–Pérot relationship (Equation (1)) [157]:

(1) $$OT_{\text{eff}}=2n_{\text{eff}}L\mathrm{Cos}\theta =m\lambda$$

where *OT_eff_* is the effective optical thickness of the thin film, *n_eff_* is its effective refractive index, *L* is the actual thickness of the film, *m* is the order of the oscillation in the interference spectrum, the maximum of which is at wavelength *λ*, and *θ* is the angle of incidence. Therefore, any change in the effective refractive index of the thin film can be detected through shifts of the interference maximum. Shifts of the interference maximum can be further resolved by establishing the *OT_eff_* of the thin film, which is calculated by applying fast Fourier transform (FFT) to the interference spectrum. The center of the resulting FFT Gaussian peak corresponds to *OT_eff_* of the Fabry–Pérot cavity. Initially, polymer and metal oxide thin films were used to create FP cavities for RIfS application [157]. However, due to their limited surface area for analyte-receptor reaction, nanoporous thin films (*i.e.*, pSi, NAA, and TiNTs) have emerged as their potential alternative [3]. In the last two decades, Sailor and co-workers exploited porous silicon as a substrate combined with RIfS to develop highly sensitive optical chemical and bio-sensors [157–159]. Due to the chemical instability of porous silicon in biological medium, the same group developed a RIfS-based sensing system using NAA as an alternative to porous silicon. NAA offers several advantages over pSi such as better chemical and mechanical stability, ease of surface modification, and more controllable and defined nanoporous structure [159]. Furthermore, NAA also acts as Fabry–Pérot cavity and thus presents well-resolved interference peaks in the RIfS spectrum [160]. These interference peaks in the RIfS spectrum allow for real-time and in-situ monitoring of binding events of biomolecules and perform label-free optical sensing (Figure 17). NAA in combination with RIfS can be used to perform highly sensitive qualitative and quantitative detection of a broad range of analyte molecules. NAA-RIfS sensing platforms have been rapidly developed to detect gases, organic molecules and biomolecules [159,161,162]. For example, Pan *et al.* prepared a NAA-RIfS sensing platform for label-free detection of complementary DNAs [163]. Alvarez *et al.* developed a label-free NAA-RIfS immunosensor to selectively monitor the capturing of target antigens by their specific antibodies [160]. Their results revealed that significant effective optical thickness change occur only when there is an exclusive and selective antigen-antibody binding reaction occurring inside NAA pores. An *et al.* reported on the optimization of interference signals of NAA based on pore widening [164]. They measured the performance of RIfS with NAA pore widened for different time intervals by monitoring the changes in the effective optical thickness of NAA after adsorption of BSA and PSA (prostate specific antigen) antigen inside NAA pores. Their results reveal that NAA with widened pores offers better sensitivity. A more comprehensive study about optimization of RIfS signal from NAA was performed by Kumeria *et al.*, where they varied parameters such as pore diameter, pore length, and surface coatings to obtain the most optimum NAA structure for RIfS based sensing platforms [160]. In other studies, Kumeria *et al.* used the optimum NAA structure for RIfS to detect volatile sulphur compounds (*i.e.*, VSCs: hydrogen sulphide gas) and hydrogen gas [165,166]. For specific detection of hydrogen sulphide gas NAA substrate were coated with a thin film of gold, whereas hydrogen gas was detected on platinum coated NAA substrates. They also showed the capability of NAA-RIfS sensing platforms to detect VSCs in human malodour using the microfluidics-based Au coated NAA-RIfS sensing system [166]. Kumeria *et al.* further combined microfluidics and NAA-RIfS to develop a microchip biosensor for detecting and quantifying circulating tumour cells (CTCs) [167]. In this system, gold coated NAA were functionalized with anti-EpCAM antibodies that specifically capture the CTCs, and changes in *OT_eff_* were used to monitor the capturing of CTCs [167]. This device was able to capture and detect CTCs in a single step without any pre-enhancement step. They also demonstrated the ability of this system to detect CTCs in human blood as well. Dronov *et al.* reported the applicability of platinum-coated NAA to act as an interferometric transducer [168]. Their results reveal that metal coatings increase the signal to noise ratio and thus improve the capabilities of the system in terms of sensing. They compared the sensing performance of NAA with porous silicon substrate, which demonstrated that NAA provides more stable signals and a better sensing performance that those of porous silicon. In a recent study, Kumeria *et al.* demonstrated the ability of mercapto-silane modified NAA substrate for an ultrasensitive sensing platforms for detecting Au(III) ions in combination with RIfS (Figure 17a) [59]. Au(III) ions have become of great relevance to clinical applications due to their widespread use in gold-based drugs and bioimaging probes [59]. For this, NAA were modified with 3-mercaptopropyl-tirethoxysilane (MPTES), which imparted the NAA with selectively gold (III) ions. The sensing performance (*i.e.*, linear range, low limit of detection, saturation concentration, and selectivity) of the NAA-RIfS sensor was assessed through a series of experiments. They also demonstrated real-life application of this NAA-RIFS sensor by detecting gold (III) ions in tap water and phosphate buffer solution (PBS).

Interestingly, NAA-RIfS platforms not only can be used to monitor filling of NAA pores and the attachment of analyte molecules to the surface functional groups but also to study the release of molecules such as drugs from NAA substrates. Using this approach, Kumeria *et al.* monitored the release of a model drug, indomethacin, in real-time under dynamic flow conditions (Figure 17b) [169]. Their results revealed that the process is diffusion-controlled and the rate of drug release depends highly on the flow rate (*i.e.*, the faster the flow rate the higher the drug release from the nanoporous platform). This approach provides more accurate information than conventional *in vitro* drug release techniques performed under static conditions. A comparative study measuring the sensing performance of RIfS and photoluminescence spectroscopy (PLS) using NAA as substrate was recently reported by Santos *et al.* (Figure 17c) [60]. First, NAA with most optimum optical signal was obtained depending on the ratio of height to width of the effective optical thickness peak for both PLS and RIfS. This most optimal NAA structure was subjected to detection of glucose and L-cysteine under non-specific and specific binding conditions, respectively. Their results suggested that the type of the analyte molecules and their binding conditions play a crucial role in the determining the sensing performance of a device. Nevertheless, NAA-PLS platforms displayed better sensing performance (*i.e.*, linearity, higher sensitivity toward analytes and lower limit of detection) for both the analytes and binding conditions than NAA-RIfS platforms. Macias *et al.* fabricated a bilayered funnel-like NAA-RIfS sensing platform [170]. This structurally engineered NAA substrate generates a complex reflectivity spectrum. Their results show that a significant enhancement of the RIfS peak intensity on coating the top surface of the bilayer-NAA with gold layer. This biosensing platform was assessed by detecting BSA molecules and the results demonstrate the ability of bilayer-NAA-RIfS platform to detect BSA only in the effective optical thickness peak corresponding to the top layer and the total layer (*i.e.*, the whole bilayer) based on size exclusion.

## 6. Future Perspective and Conclusions

We believe that there is still a huge potential for further improvement of basic properties of NAA, which can be utilized for broad and multidisciplinary research fields as materials science, nanotechnology, optics, electrochemistry, cell biology and medicine. Over the last few decades, the different approaches have made it possible to design the structure of NAA, resulting in better control over pore diameter, length, porosity and organization of pores. However, the real understanding of the self-organizing anodization process on aluminum is not complete. Thus, to generate and extend the range of NAA structures available, it is necessary to investigate this phenomenon intensely. For its wider commercial application, the cost is a critical factor for NAA to be used as a consumable, however, the cost of high purity aluminum may be bottom line in this scenario. In terms of surface chemistry, the surface properties of NAA can be finely tuned in limited ways due to inherent properties of the process used to functionalize NAA (*i.e.*, limited coverage, stability, and terminal groups). In the future we may expect more versatile surface functionalization process to achieve chemistries unimaginable with present techniques and several research groups are already working towards it.

This review has summarized and presented the recent progresses in surface functionalization and modification of NAA and the developments for its use in localized plasmon and reflective interference based sensing systems. We have presented a detailed description about structure and surface modifications of NAA. We have also provided detail of the fundamental aspects of LPR and RIfS in combination with NAA platforms along with the most relevant concepts and examples of devices reported based on NAA and these optical techniques. Due to the advanced properties of NAA, it has emerged as a highly attractive alternative for development of broad range stable and functional sensing devices for clinical, industrial and environmental analyses. The ability to modify the surface of NAA with a wide range of functionalities to endow it with desired selectivity and specificity towards target analytes has pushed NAA for applications in several fields. We believe that future developments in structural engineering and chemical modifications of NAA will yield more innovative NAA-based sensing systems. Furthermore, a number of methods to structurally engineer the NAA make it further attractive nanostructures for development of future optical sensing devices. However, this review has demonstrated that there is still plenty of advances and developments to be made in the field of structural engineering, surface modification, and NAA-LPR and NAA-RIfS based sensing platforms.

## Figures and Tables

**Table 1.** Summary of the self-organizing conditions and pore diameters obtained during MA and HA regimes for the most commonly used electrolytes in NAA fabrication.

**Acid Electrolyte**	**Mild Anodization**				**Hard Anodization**			

	***T* (°C)**	***V* (V)**	**D_p_ (nm)**	**Ref.**	***T* (°C)**	***V* (V)**	**D_p_ (nm)**	**Ref.**
H_2_SO_4_ 0.3 M	5	25	25	[15]	0	40	30	[23]
H_2_C_2_O_4_ 0.3 M	5–8	40	30	[14]	0–1	140	50	[8]
H_3_PO_4_ 0.1 M	0–1	195	160	[16]				
H_3_PO_4_:C_2_H_5_OH: H_2_O (1:20:79 v/v)					−10 to 0	195	80–140	[9]

**Table 2.** Summary of the variety of silanes used to functionalize NAA and their applications.

**Types**	**Silane Name**	**Modification Method**	**Application(s)**	**Ref.**
Amino-silane	Aminopropyl-triethoxy silane	Solution based	Immobilization	[29,58]
PEG-silane	mPEG-silane	Solution based	Hydrophylization	[65–67]
Fluorinated-silane	perfluoroalkyl-silanes	Solution based	Transporting and separation	[5]
Mercapto-silane	Mercaptopropyl-triethoxy silane	CVD based	Sensing	[59]

## Acronyms

Nanoporous anodic alumina: NAA
Surface plasmon resonance: SPR
Reflectometric interference spectroscopy: RIfS
Porous silicon: pSi
Titania nanotubes: TiNTs
Pore diameter: D_p_
Inter-pore distance: D_int_
Pore length: L_p_
Oxide barrier layer thickness: L_b_
Mild anodization: MA
Hard anodization: HA
Photoluminescence: PL
Effective optical thickness: OT*_eff_*
Chemical vapor deposition: CVD
Atomic layer deposition: ALD
Surface enhanced Raman scattering: SERS
Water contact angle: WCA
Carbon nanotubes: CNTs
Self-assembled monolayers: SAMs
Layer-by-Layer: LbL
Polyelectrolyte: PE
Silica nanotubes: SNTs
Distributed Bragg reflector: DBR
Rugate filters: RF
Localized surface plasmon resonance: LSPR/LPR
Waveguide: WG
Limit of detection: LOD
Fabry–Pérot: FP
Fast Fourier transform: FFT
Volatile sulfur compounds: VSCs
Circulating tumor cells: CTCs
Phosphate buffer solution: PBS
